# *Aspergillus fumigatus*: a rare cause of peritonitis in a peritoneal dialysis patient (a case report)

**DOI:** 10.11604/pamj.2022.43.35.32583

**Published:** 2022-09-21

**Authors:** Amori Engelbrecht, Roland van Rensburg, Dawood da Costa, Mogamat-Yazied Chothia

**Affiliations:** 1 Department of Medicine, Division of Nephrology, Faculty of Medicine and Health Sciences, Stellenbosch University, Cape Town, South Africa,; 2 Department of Medicine, Division of Clinical Pharmacology, Faculty of Medicine and Health Sciences, Stellenbosch University, Cape Town, South Africa,; 3 Department of Pathology, Division of Medical Microbiology and Immunology, Faculty of Medicine and Health Sciences, Stellenbosch University and National Health Laboratory Service, Tygerberg Hospital, Cape Town, South Africa

**Keywords:** Fungal, *aspergillus fumigatus*, dual infection, peritoneal dialysis, case report

## Abstract

Although fungal organisms remain an uncommon cause of peritonitis in patients undergoing peritoneal dialysis, it carries significant morbidity and mortality. We describe a 36-year-old woman who presented with peritoneal dialysis-related peritonitis caused by dual infection with Staphylococcus caprae and Aspergillus fumigatus and discuss the challenges that were confronted regarding microbiological diagnosis and the selection of optimal antifungal treatment with subsequent successful resumption of peritoneal dialysis.

## Introduction

Although fungal organisms remain an uncommon cause of peritonitis in patients undergoing peritoneal dialysis (PD) [1] it is associated with high rates of hospitalization, technique failure, and death [2]. Non-albicans Candida species (spp.) are the most common cause of fungal peritonitis, while *Aspergillus spp* are rare, comprising only 2% to 5% of all fungal peritonitis cases [3]. Challenges regarding microbiological diagnosis and selecting the optimal antifungal treatment for *Aspergillus*-related peritonitis exist. Here we present a challenging case of PD-related bacterial and fungal peritonitis caused by *Staphylococcus caprae* and *Aspergillus fumigatus*.

## Patient and observation

**Patient information:** a 36-year-old woman with end-stage kidney disease was receiving PD as her kidney replacement therapy modality for nine months without any major events; however, during the index presentation she had diffuse abdominal pain, constipation, lower limb swelling and associated cloudy effluent for five days. She had a history of geophagia. She lived in informal housing, slept on soil-covered flooring, and often had to perform her PD exchanges under poor conditions.

**Clinical findings:** on examination her abdomen was distended and diffusely tender with no features of tunnel or exit site infection. Blood pressure was 97/58 mmHg, pulse rate of 80 beats per minute and body temperature of 35.3°C. Hydration was normal, and she had conjunctival pallor.

**Timeline of episode:** following one week of empiric outpatient therapy consisting of intraperitoneal (IP) vancomycin 1.5 g every five days and IP amikacin 100 mg with every night-time exchange, the patient´s abdominal pain and cloudy peritoneal effluent were ongoing. She was hospitalized and repeat blood and peritoneal effluent samples were sent for culture. Intraperitoneal vancomycin and amikacin were continued.

**Figure 1:**
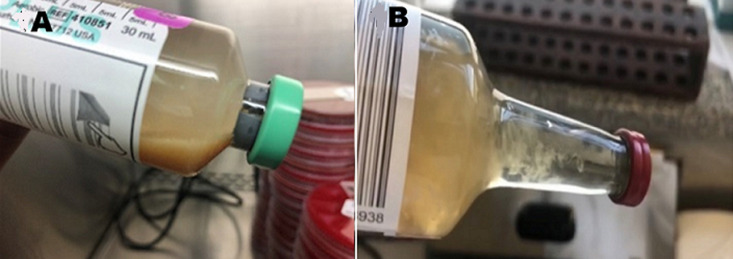
A) Bact/alert® fan® aerobic plus; B) bd Bactec® myco/F lytic culture vial

**Figure 2:**
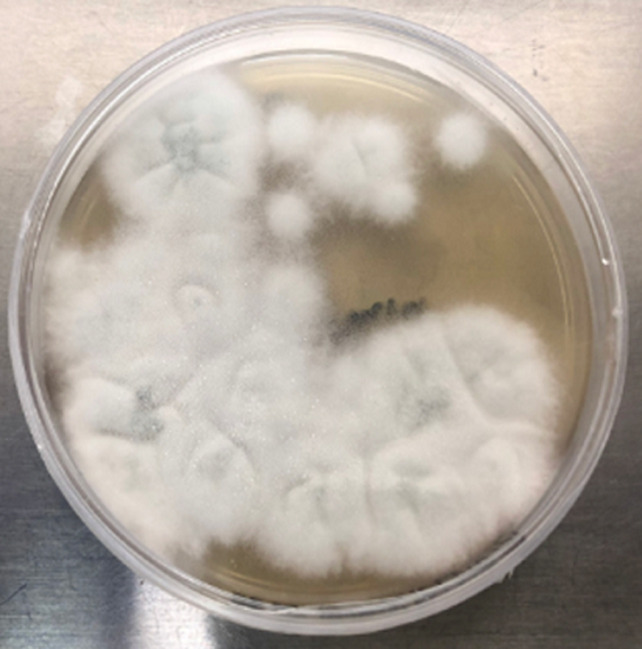
saboraud's dextrose agar following overnight incubation at 35°C carbon dioxide-enriched incubation

**Figure 3:**
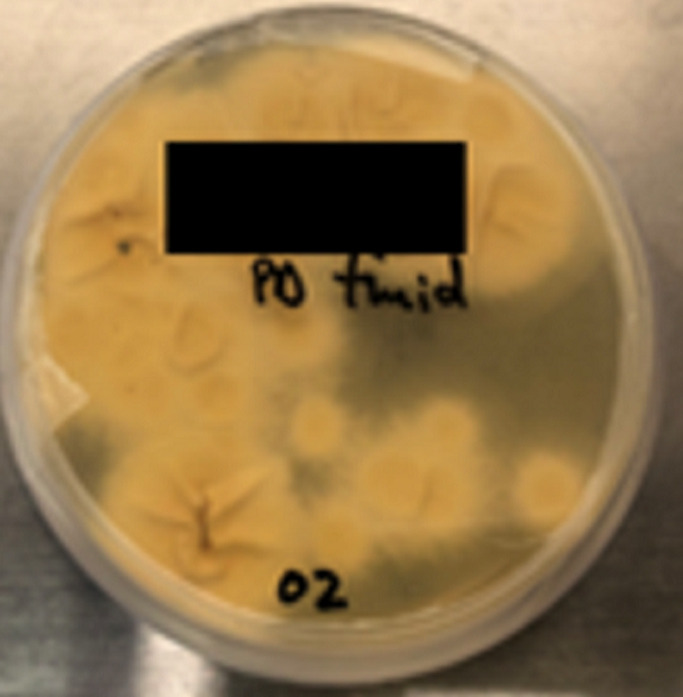
saboraud's dextrose agar following overnight incubation at 37°C

**Figure 4:**
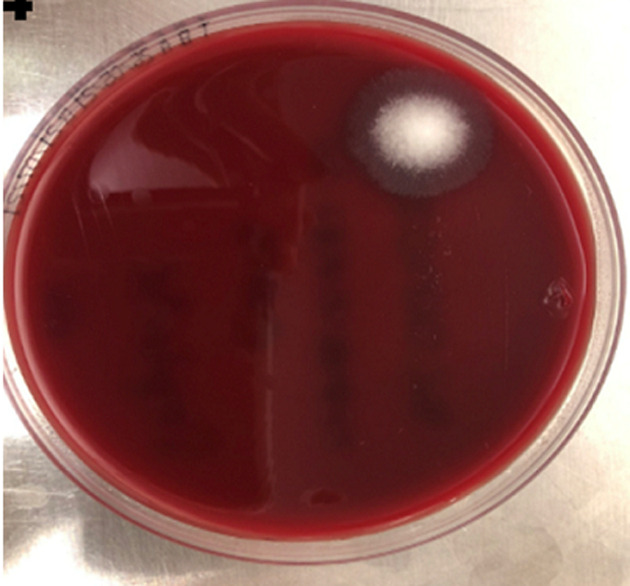
two percent (2%) tryptose blood agar following overnight incubation at 37°C

**Figure 5:**
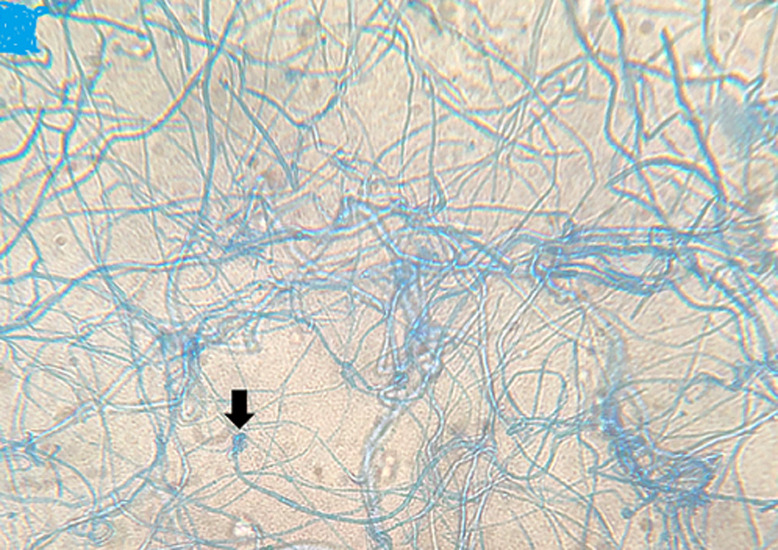
lactophenol cotton-blue stain at 400 times magnification

**Diagnostic assessment:** initial peritoneal effluent sample revealed a nucleated cell count of 1.31 x 10^9^/L (normal range: < 0.1x10^9^/L) with a differential cell count of 68% neutrophils and 11% lymphocytes, supporting a diagnosis of PD-related peritonitis. The initial peritoneal fluid culture flagged positive after 16- hours of incubation. No bacteria or yeasts were seen on gram stain; however, on subculture of the peritoneal fluid, fungal-like elements were macroscopically visible in the culture bottle (Figure 1A, Figure 1B); and on microscopy hyphal elements suggestive of fungi were seen on 10% potassium hydroxide wet preparation and subsequently *Aspergillus fumigatus* was isolated (Figure 2, Figure 3, Figure 4, Figure 5). Since *Aspergillus spp*. are ubiquitous organisms, this was initially considered to be a contaminant; however, during her hospitalization repeat peritoneal effluent culture was performed. After 48-hours incubation, a coagulase negative staphylococcus was isolated, *Staphylococcus caprae* along with *Aspergillus fumigatus*.

**Diagnosis:** dual infection of *Staphylococcus caprae* and *Aspergillus fumigatus-*related peritonitis in a peritoneal dialysis patient.

**Therapeutic interventions:** the patient was initiated on oral voriconazole 400 mg 12-hourly for one day as a loading dose, followed by 200 mg 12-hourly for six weeks, while IP vancomycin was continued and the Tenckhoff catheter was removed.

**Follow-up and outcome of interventions:** the patient demonstrated clinical improvement and peritoneal effluent cultures were negative at two and three weeks after initiation of oral voriconazole. Due to limited availability of hemodialysis slots as well as the patient´s preference for PD as a dialysis modality, PD was reinitiated. Two months after resumption of PD, the patient was doing well.

**Patient perspective:** the patient preferred PD as her dialysis modality and was elated when the attempt at resumption of PD was successful.

**Informed consent:** the patient gave written, informed consent and the Human Research Ethics Committee at Stellenbosch University gave permission for publication.

## Discussion

*Aspergillus spp* are ubiquitous organisms, commonly found in soil and decaying matter [4], with *Aspergillus fumigatus* and *Aspergillus niger* most frequently isolated in *Aspergillus*-related PD peritonitis [2]. Although it rarely causes peritonitis in the PD population, *Aspergillus* is associated with mortality rates of up to 50% [5]. This is attributed to delays in diagnosis, failure to remove the Tenckhoff catheter, and limited therapeutic options [6]. Confirmation of invasive *Aspergillus spp* infection is challenging due to potential specimen contamination, frequent respiratory colonization, potential laboratory contamination of culture specimens and suboptimal sensitivity for isolation of *Aspergillus spp* using routine culture methods [7]. Subsequently, it has been recommended that the diagnosis should be confirmed by more than one positive culture [3], or immunodiagnostic testing of the effluent [6]. Traditional risk factors for the development of fungal PD-related peritonitis include recent exposure to corticosteroid therapy, immunosuppression, prolonged use of broad-spectrum antibiotics, recent bacterial peritonitis, and recent hospitalization [6]. Notably, we did not identify these risk factors in our patient. Since *Aspergillus fumigatus* is ubiquitous in soil, we postulated an association between the patient´s geophagia and poor home circumstances as predisposing factors to fungal colonization and subsequent PD-related *Aspergillus* peritonitis. Evidence-based treatment guidelines for *Aspergillus* peritonitis are limited. The International Society of Peritoneal Dialysis recommends immediate catheter removal when there is high clinical suspicion of fungal peritonitis with temporary transition to hemodialysis and treatment with an antifungal agent for 4-6 weeks [2]. It was postulated that the peritoneal dialysis catheter may not only act as a portal of entry for infection, but also as a microbial reservoir, making catheter removal imperative to salvage PD as a future modality [6].

*S. caprae* is a coagulase-negative staphylococcus and this group of bacteria is responsible for half of all gram-positive peritonitis cases in PD [8]. Since our patient demonstrated clinical improvement with clearing of the cloudy effluent, the initial culture was not considered a contaminant, and continuation of vancomycin seemed reasonable. Antifungal drugs effective against *Aspergillus spp* include the polyenes (amphotericin B), azoles (itraconazole and voriconazole), and echinocandins (caspofungin, micafungin and anidulafungin) [3]. Both the deoxycholate and liposomal formulations of amphotericin B have poor penetration into the peritoneal cavity when administered systemically [9] and therefore are not recommended. Intraperitoneal administration is also not recommended, due to the elevated risk of local effects such as chemical peritonitis and adhesion formation, and loss of a port of access when the catheter is removed as part of source control [10]. Data on the efficacy of the echinocandins in *Aspergillus* peritonitis are sparse and mostly anecdotal [11]. Data regarding itraconazole are also limited in *Aspergillus* peritonitis, and where available for invasive *Aspergillosis*, was shown to be only moderately effective [12]. Voriconazole is currently recommended as the best available treatment for *Aspergillus*-related PD peritonitis, because of excellent penetration into the peritoneal cavity [13] and has been used with good success in reported cases [14]. Patients with *Aspergillus* peritonitis are rarely able to resume PD, and often require a permanent transition in modality [14]. In a recent systematic review, 82% of patients who survived *Aspergillus* peritonitis were switched to hemodialysis [3]. The low rate of PD resumption has been ascribed to delay in diagnosis and extensive peritoneal cavity adhesions [14].

## Conclusion

*Aspergillus fumigatus* is a rare cause of peritonitis in PD. Diagnosis and management may be challenging especially when dual infection is present and requires a multi-disciplinary approach. Although it frequently requires a permanent transition in dialysis modality, successful resumption of PD may be possible.
